# Enhancement of Cerebral Oxygenation by Lower-Limb Neuromuscular Electrical Stimulation in Stroke Survivors

**DOI:** 10.1007/s10439-026-04209-3

**Published:** 2026-06-25

**Authors:** Anirban Dutta, Yashika Arora, Sandra Leason, Allam Harfoush, Kausik Chatterjee

**Affiliations:** 1 Department of Metabolism and Systems Science, School of Medical Sciences, College of Medicine and Health, University of Birmingham, Birmingham, B15 2TT UK; 2 All India Institute of Medical Sciences, New Delhi, India; 3 Countess of Chester Hospital NHS Foundation Trust, Chester, UK

**Keywords:** Ischaemic stroke, Rehabilitation, Neuromuscular electrical stimulation, Cerebral perfusion, Functional near-infrared spectroscopy, Cardiovascular systems modelling

## Abstract

**Purpose:**

Stroke remains a leading cause of death and long-term disability with few non-pharmacological interventions available to enhance cerebral perfusion during recovery. Neuromuscular electrical stimulation (NMES) of the common peroneal nerve (CPN) may augment venous return and cardiac output via activation of the skeletal muscle pump and autonomic reflexes thereby supporting cerebral blood flow. This study aimed to evaluate the effects of low-frequency (1 Hz) CPN-NMES on cortical oxygenation in ischaemic stroke survivors and to mechanistically interpret these effects using cardiovascular computational modelling.

**Methods:**

In the Phase 1 RETRAIN study (NCT06614400), eighteen individuals with ischaemic stroke underwent transcutaneous CPN-NMES delivered at 1 Hz (1 pulse per second) using a wearable device at graded stimulation intensities across three postures (supine, semi-supine, and sitting). Cortical oxygenation was continuously monitored using functional near-infrared spectroscopy (fNIRS). A lumped-parameter cardiovascular model was used to simulate NMES-induced changes in venous return, cardiac output, and systemic haemodynamics. Linear mixed-effects models analysed experimental data, with transcutaneous carbon dioxide included as a covariate to account for cerebrovascular reactivity.

**Results:**

CPN-NMES elicited significant, dose-dependent increases in cortical oxygenation. Effects were amplified in upright postures and in participants with larger infarct volumes (>5 cm). Computational modelling confirmed that 1 Hz CPN-NMES enhances venous return and cardiac output while maintaining haemodynamic stability. Sex-specific cardiovascular adaptations were observed with females demonstrating greater heart rate-mediated responses.

**Conclusion:**

Low-frequency CPN-NMES enhances cortical oxygenation in stroke survivors through posture-, lesion-, and sex-dependent cardiovascular mechanisms. Wearable CPN-NMES represents a promising, non-invasive adjunct to stroke rehabilitation that warrants further investigation in larger controlled trials.

## Introduction

Stroke remains a leading cause of death and adult disability worldwide, with ~ 15 million cases annually (110,000 in the UK) and an enormous healthcare burden (~ £26 billion per year in the UK alone) [1]. Beyond the immediate cerebral injury, acute ischaemic stroke can precipitate serious cardiac complications–a phenomenon termed stroke–heart syndrome (SHS) [2]. Introduced in 2018 as an integrated concept, SHS encompasses the spectrum of cardiovascular disturbances within ~ 30 days post-stroke, including myocardial injury, acute coronary syndromes, arrhythmias, and heart failure. Notably, 10–20% of stroke patients experience such cardiac complications in the first few days. This brain-heart interplay presents a dual threat, i.e. cerebral ischaemia is exacerbated by compromised cardiac output and dysregulated autonomic control complicating both acute management and recovery. In a cohort of 15,054 ischaemic stroke patients, 11.8% developed SHS, typically within 2 days of onset [3]. However, SHS occurring between 10 and 30 days carried nearly double the 90-day mortality risk compared to SHS within the first 3 days (adjusted heart rate 1.84, 95% CI 1.36–2.49). Amongst manifestations, acute myocardial injury, heart failure/ left ventricular dysfunction, and atrial fibrillation/flutter were linked to the highest 90-day mortality. Overall, SHS substantially increases post-stroke death risk, with delayed onset particularly dangerous. Early post-stroke care thus faces a critical challenge, i.e. how to optimally restore and maintain cerebral perfusion in the context of unstable systemic haemodynamics and autonomic imbalance. Even when arterial recanalization is achieved (e.g. via thrombolysis or thrombectomy), microvascular perfusion in the penumbra can remain suboptimal due to impaired autoregulation, “no-reflow” phenomena, or systemic factors like low cardiac ejection fraction [4]. Inadequate cerebral autoregulation and neurovascular coupling during this vulnerable period can accelerate infarct expansion and worsen outcomes [5]. A recent study found that cerebral autoregulation is impaired in acute ischaemic stroke with patients showing lower autoregulation indices than controls and a novel early transient peak in critical closing pressure (CrCP) dynamics [6]. Furthermore, loss of autonomic homeostasis after stroke (characterized by excessive sympathetic and reduced parasympathetic activity) contributes to arrhythmias and cardiac dysfunction which in turn reduce cerebral perfusion—a vicious cycle of SHS.

Non-invasive peripheral electrical stimulation aims to improve cerebral blood flow and cardiac function by activating peripheral nerves or muscles rather than directly stimulating the brain [7]. Human and animal studies show non-invasive peripheral electrical stimulation techniques can boost cerebral oxygenation, Internal Carotid Artery (ICA) blood flow, though results are inconsistent and protocol dependent [7]. Studies use varied protocols with frequencies from 2 to 120 Hz, pulse widths of 50–400 µs, and intensities ranging from mild sensory currents to strong muscle contractions. Nerve excitability varies with axonal properties such as membrane conductances and strength–duration characteristics. The strength–duration time constant, a measure related to chronaxie, is reliably shorter in large, myelinated motor axons than in cutaneous sensory afferents in humans, reflecting differences in voltage-dependent Na⁺ conductances at the node of Ranvier [39]. This phenomenon has been attributed to a greater persistent (non-inactivating) Na⁺ conductance in sensory fibres, which prolongs the effective membrane time constant and increases excitability relative to motor fibres [40, 41]. The stronger persistent Na⁺ current in cutaneous afferents likely underlies their longer strength–duration characteristics and greater propensity for ectopic activity compared with motor axons [42]. Sessions typically last 5–30 minutes, applied once or repeatedly over days.

Main techniques include neuromuscular electrical stimulation (NMES), transcutaneous electrical nerve stimulation (TENS), and functional electrical stimulation (FES). Experimental studies across diverse populations have demonstrated that calf NMES can significantly increase venous blood velocity and volumetric flow in the deep veins of the lower limb [8]. Early proof-of-concept studies in healthy volunteers demonstrated that calf NMES can acutely enhance venous return [9]. NMES applied to the calf muscles produces immediate increases in popliteal and femoral vein blood flow [10]. One report [11] noted median popliteal velocities rising from ~ 7 cm/s at rest to ~ 70 cm/s with a voluntary calf contraction and to ~ 13 cm/s with electrically induced contraction in standing subjects. Although voluntary effort generated the highest flow (since maximal effort can be greater than tolerable NMES intensity), NMES still produced a significant haemodynamic gain over resting conditions where high-frequency, high-intensity recruit more muscle fibres, yielding greater venous outflow [12]. Over a 30-minute NMES session (36 Hz, pulse width of 350 µs), healthy subjects showed sustained venous ejection volumes indicating no fatigue-induced drop-off in performance [13]. In fact, as users habituated to the stimulation over days, they could comfortably tolerate higher intensities, leading to approximately double the venous ejected volume after one week of daily NMES compared to the first day [13]. For NMES (4 Hz biphasic pulse width, 250 µs) applied to large lower body muscles [14] and using ultrasound flow measurements in major cerebral arteries, authors found ICA blood flow increased by ~ 12% during calf/thigh NMES (from ~ 330 mL/min at rest to ~ 371 mL/min). In contrast, vertebral artery flow (posterior circulation) did not change appreciably. The rise in ICA flow suggests improved perfusion to anterior brain regions during NMES. Notably, there was a strong linear correlation between the increase in end-tidal CO_2_ (a measure of carbon dioxide retention) and the increase in ICA flow during NMES (*R* = 0.74). This implies the mechanism was partly CO_2_-mediated cerebral vasodilation because the subjects were not volitionally exercising, their ventilation did not initially rise to offset increased CO_2_ production by the stimulated muscles, leading to mild hypercapnia that dilated cerebral vessels. Therefore, NMES, whether low-frequency twitch contractions (e.g. 4 Hz, 250 µs, 20 min) that can raise carotid blood flow by ~ 12% in healthy adults [14] or high-frequency, high-intensity quadriceps protocols (100 Hz, 400 µs, up to 50 mA) that can even boost Brain-Derived Neurotrophic Factor and cognition [15], can acutely benefit brain function. Then, FES where NMES is patterned for functional tasks, particularly FES-assisted cycling elicits cerebral blood flow and oxygenation increases comparable to voluntary exercise [16], supporting its role in early post-stroke rehabilitation. In a published study [17], participants sat on an ergometer with standardized trunk (90°) and knee (95°) angles whilst isometric NMES was applied bilaterally to the quadriceps. Here, 40 Hz NMES with a pulse width of 400 μs and a duty cycle of 6-sec ON and 6-sec OFF significantly increased middle cerebral artery blood flow velocity (MCA) and prefrontal oxyhaemoglobin with no changes in posterior cerebral artery velocity (PCA) or other oxygenation markers. NMES also elevated heart rate and cardiac output whilst mean arterial pressure rose similarly in both NMES and control sessions. These healthy human findings confirm that brief stimulation bouts (5 Hz with intensity set to 10% below pain threshold) can eject a substantial volume of blood from the calf veins likely due to enough refill time at lower frequencies. Here, belt electrode skeletal NMES can stimulate large muscle groups in the lower limbs [18] and evidence from lower-limb NMES/FES indicates its potential to improve cerebral perfusion [7, 12, 17]. In the study in healthy young men [18], belt electrode skeletal muscle electrical stimulation (250 μs pulses at 4 Hz for 20 min) acutely increased heart rate, cardiac output, mean arterial pressure and blood flow in the femoral artery; however, it did not change stroke volume (the amount of blood pumped per heartbeat), total peripheral resistance (afterload) or the artery’s diameter. Then in a randomized crossover study [19], both TENS and NMES significantly increased popliteal vein flow volume and peak velocity in healthy subjects compared to sham with TENS producing a larger relative increase in flow volume than NMES. Specifically, in young healthy adults, TENS increased popliteal vein time-averaged flow volume by more than 220% compared to baseline. In the same study, time-averaged flow volume in the popliteal vein rose ~ 37% with NMES. Here, TENS and NMES both used symmetrical biphasic square waveforms with a 350 µs phase duration but differed in frequency with 5 Hz for TENS and 35 Hz for NMES. Then, cervical TENS has been tested at frequencies from 2 up to 120 Hz with pulse widths ~ 50–200 μs yield mixed results, e.g. in healthy adults it does not alter MCA flow [20] likely due to the intact cerebral autoregulation but in patients with vasospasm it improves cerebral blood flow [21] and in low-frequency sub-threshold settings can modestly reduce carotid velocity [22]. Overall, NMES (4–100 Hz, 250–400 µs, 6–50 mA), TENS (2–120 Hz, 50–200 µs, sensory to near-pain level), and FES (20–50 Hz, 300–450 µs, 15–60 min) demonstrated parameter- and context-dependent capacity to augment cerebral perfusion and oxygenation [7].

We hypothesized based on prior studies that low-frequency 1 Hz NMES targeting the common peroneal nerve (CPN) may more effectively enhance venous return and cardiac preload thereby modulating cardiac output, blood pressure, and cerebral perfusion under impaired autoregulation in early stroke [23, 24]. Here, 1 Hz NMES of CPN targeted the lower-limb venous return system as a coordinated network of collapsible veins whose function is critically dependent on external mechanical forces and competent venous valves. Recent findings by Juthberg and colleagues [25] demonstrated that 1 Hz NMES via sock-integrated electrodes enhances peripheral haemodynamics more effectively and comfortably than 36 Hz stimulation. Building upon this, we proposed that such low-frequency (closely matching resting heart rate) NMES may also positively influence cerebral oxygenation [12]. This hypothesis is grounded in the physiological interplay between peripheral venous return and cerebral blood flow at lower frequencies especially considering the muscle pump’s role in circulatory dynamics [26]. By delivering electrical pulses at 1 Hz to the CPN and associated lower-limb musculature, NMES elicits rhythmic muscle contractions that activate the skeletal muscle pump thereby enhancing venous return and increasing cardiac preload. In the upright posture, hydrostatic pressure causes substantial venous distension, but this is physiologically counteracted by three integrated pumping mechanisms, the calf muscle pump, the distal calf pump, and the plantar (foot) venous pump. During muscle contraction, intramuscular pressure rises sharply, expelling blood proximally into the popliteal vein, whilst valve competence prevents retrograde flow; during relaxation, pressure falls and the deep veins refill from superficial and arterial sources. Experimental studies across diverse populations have already demonstrated that NMES can significantly increase venous blood velocity and flow for preventing venous thromboembolism (VTE) [8]. The geko™ system has FDA 510(k) clearance for short-term post-surgical neuromuscular stimulation as an adjunct to the prevention of venous thrombosis; however, broader use of NMES for routine VTE prophylaxis remains investigational [27]. The device delivers electrical stimulation to the CPN, producing rhythmic ankle dorsiflexion that mechanically activates the lower-limb venous pump system, thereby enhancing venous return, with reported secondary improvements in cerebral oxygenation [12].

Senin-Camargo and colleagues reported a study that provided a direct comparison of venous haemodynamic responses to TENS, NMES, and sham stimulation applied to the soleus muscle [19]. The study compared sustained-contraction NMES with low-frequency twitch stimulation delivered using a TENS device; although labelled as TENS, the latter protocol produced visible muscle contractions and therefore functioned as motor-level stimulation rather than purely sensory TENS. In our own healthy human study, we measured regional blood volume/oxygenation changes in both muscle and brain during NMES with the geko™ system [12]. We combined muscle NIRS (mNIRS) and functional NIRS (fNIRS) in healthy subjects performing calf exercise (study 1) and NMES (study 2). In the volitional tiptoe task without NMES (study 1) [28], healthy participants performed movements at 10, 30, and 50 tiptoe movements per minute. Results showed that muscle activity (electromyogram power) increased with tiptoe movement rate whilst muscle blood volume (total haemoglobin, HbT) decreased significantly likely due to reduced venous refill time at higher tiptoe rates. Notably, females exhibited a greater reduction in muscle blood volume than males suggesting gender-related differences in filling dynamics [29]. Computational study using fluid-structure interaction (FSI) modelling [28] investigated NMES-induced haemodynamic changes under varying physiological conditions. FSI modelling indicated that stronger (higher frequency and intensity) vessel contractions and lower blood viscosity enhance venous outflow whilst lower frequencies allow more refill time which vary across individuals [29]. Therefore, in the NMES study (study 2), the geko™ device was used to stimulate the CPN at 1 Hz across 11 increasing stimulation intensities. Our results showed that muscle blood volume (HbT) in the calf, measured via mNIRS, decreased significantly (*p* < 0.01) with higher stimulation intensities [12] consistent with enhanced venous ejection. Additionally, cerebral blood volume measured by fNIRS increased significantly (*p* < 0.01) with stimulation intensities [12] indicating a neurovascular response. Then, differences in muscle haemodynamics were also observed between males and females suggesting gender-based variability [28].

In line with our healthy human study [12], the intended role of the NMES intervention is acute physiological support of venous return and cardiac preload to transiently stabilise systemic haemodynamics and thereby support cerebral perfusion during the vulnerable post-stroke period. Consistent with this feasibility focus, the first phase of the RETRAIN trial (NCT06614400) was designed to evaluate how varying geko™ NMES intensities and body postures influence cortical oxygenation in patients with ischaemic stroke. The fNIRS was used as the primary brain monitoring modality, and comparable numbers of male and female participants were included to enable exploration of gender-based differences in physiological response. The study investigated how to optimize body posture and stimulation parameters for repurposing the geko™ NMES device to enhance cerebral oxygen delivery up to six months post-stroke. Body posture is crucial since clinical data has shown that autonomic dysfunction is common and can persist up to six months post-stroke [30]. Moreover, postural changes from supine to sitting and standing has been shown to significantly alter cerebral blood flow, critical closing pressure, and resistance area product, highlighting posture-dependent effects on dynamic cerebral autoregulation [31]. The second phase of the RETRAIN trial (NCT07197996) is now evaluating the geko™ device against intermittent pneumatic compression during the hyperacute phase (within 36 hours) to support cerebral oxygenation.

In this manuscript, we report results from Phase 1 of the RETRAIN trial, employing computational modelling to characterise cerebral oxygenation dynamics across postural conditions (supine vs. sitting) and graded stimulation intensities. Phase 1 was explicitly designed as a feasibility study rather than for confirmatory hypothesis testing. Accordingly, the sample size was determined pragmatically to generate parameter estimates to inform the design, power calculations, and optimisation strategy for Phase 2 (ClinicalTrials.gov ID: NCT07197996). We also present lumped-parameter cardiovascular modelling results to explain gender differences in autonomic functions and blood pressure regulation under NMES [32, 33]. Representing vascular compartments as electrical circuit analogues enabled simulation of both peripheral and central haemodynamic responses forming the basis for future model-based control strategies that could allow adaptive NMES. Complementing the modelling, RETRAIN experimental results reinforced 1 Hz NMES as a feasible intervention to improve both cerebral and systemic circulation. In the context of unstable systemic haemodynamic and autonomic imbalance where critical closing pressure strongly influences cerebral blood flow [6], our computational findings and clinical observations support further investigation of NMES as a personalized, physiologically informed therapy to augment venous return and cardiac preload, thereby stabilizing cardiac output, blood pressure.

## Materials and Methods

### Study Design

**Fig. 1:**
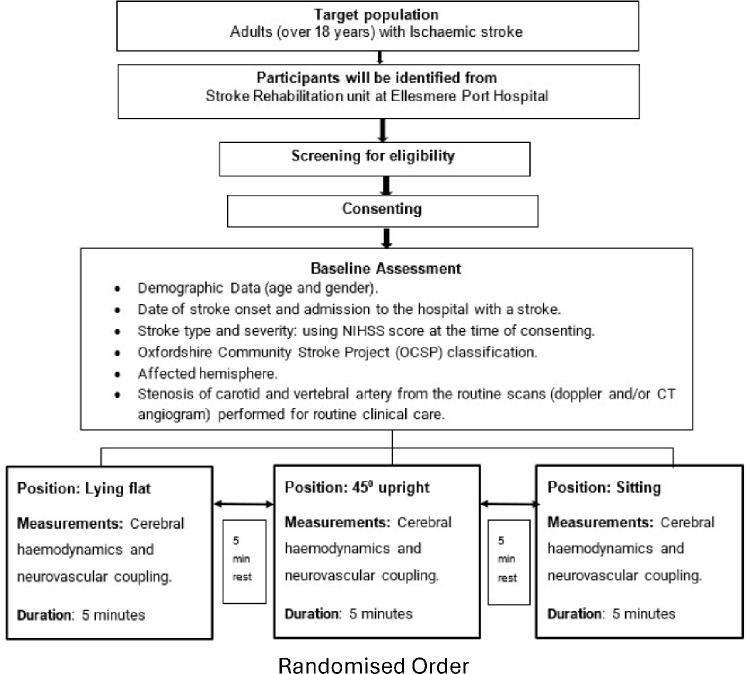
RETRAIN trial flow chart Phase 1

The RETRAIN Phase 1 trial (NCT06614400) was a prospective observational study carried out at a single site at the Stroke Rehabilitation Unit at Ellesmere Port Hospital in Chester, UK. The study was developed in accordance with Good Clinical Practice standards and the principles outlined in the Declaration of Helsinki. Ethical approval was obtained before participant enrolment, and all individuals provided written informed consent prior to taking part. A flowchart illustrating the trial process is presented in Fig. 1.

### Participants

The study enrolled adults aged 18 years and above who had experienced a confirmed ischaemic stroke, verified by either computed tomography (CT) or magnetic resonance imaging (MRI), and were beyond the acute phase specifically more than seven days post-stroke. To be included, participants needed to safely transition, with assistance, into three designated positions, i.e. lying flat (supine), reclined at a 45-degree angle (semi-supine), and sitting upright. Additionally, they had to be receiving NMES using the geko™ device as part of their standard care for VTE prevention.

Exclusion criteria covered a wide range of conditions such as recent strokes within the past seven days, a history of transient ischaemic attack (TIA), epilepsy, peripheral nerve disorders, recent lower-limb amputations, the use of other neuromodulation technologies, or the inability to give informed consent due to cognitive or communication challenges.

### Procedures

At recruitment, participants underwent comprehensive baseline evaluations, which included demographic information (such as age and gender), assessment of stroke severity using the National Institutes of Health Stroke Scale (NIHSS), and review of relevant vascular imaging results (e.g. Doppler ultrasound or CT angiography) obtained during standard clinical care.

Eligible individuals were selected by an experienced stroke physician. Once appropriately positioned, fNIRS probes from the NIRSport2 system (NIRx Medical Technologies, LLC, Glen Head, NY, USA) were applied bilaterally to the sensorimotor regions of the scalp. Special attention was given to avoid hair-covered or sinus areas, ensuring optimal signal acquisition. Probes were affixed using double-sided adhesive pads and elastic bands to maintain stable contact throughout data collection.

The intervention involved transcutaneous NMES using the geko™ T-3 device (Firstkind Ltd, UK) targeting the common peroneal nerve (CPN) in both legs. The geko™ T-3 device delivers biphasic, charge-balanced electrical pulses to the CPN at a fixed frequency of 1 Hz. Stimulation intensity is adjustable via multiple selectable current output levels (27 mA, 38 mA, 54 mA) with pulse widths modifiable over a range of approximately (±10%) 35–560 µs (see Fig. S8, Online Resource SupplementaryFigures.zip). Parameters are titrated from 11 stimulation modes (35, 50, 70, 100, 140, 200, 280 μs @27 mA, 280 & 400 μs @38 mA, 400, 560 μs @54 mA) to produce visible ankle dorsiflexion, generating continuous rhythmic twitch contractions (called “VTE optimal”). This action is intended to enhance lower-limb venous return. Each participant received stimulation at five different intensity levels, i.e., “VTE optimal”, one level above optimal, and three progressively lower settings. These stimulations were administered across three distinct body positions, i.e. laying flat (supine), reclined at a 45-degree angle (semi-supine), and seated upright. Each stimulation session lasted five minutes and was followed by a five-minute rest interval to mitigate potential carry-over effects.

Throughout the protocol, physiological parameters were closely monitored. Real-time transcutaneous carbon dioxide (tcPCO_2_) levels were measured every second (1 Hz) using the Sentec Digital Monitoring System (Timik Medical OY, Denmark) alongside regular blood pressure and environmental temperature to minimize confounding factors. At session end, probes were removed and data were de-identified and queued for single-blind analysis.

### Safety Monitoring

Safety monitoring was conducted throughout the study with structured procedures in place for documenting adverse events, serious adverse events, and adverse device effects. Instances of systemic hypotension, tachycardia, and any complications related to the device were carefully tracked and addressed following established clinical guidelines.

### fNIRS Measurement

**Fig. 2:**
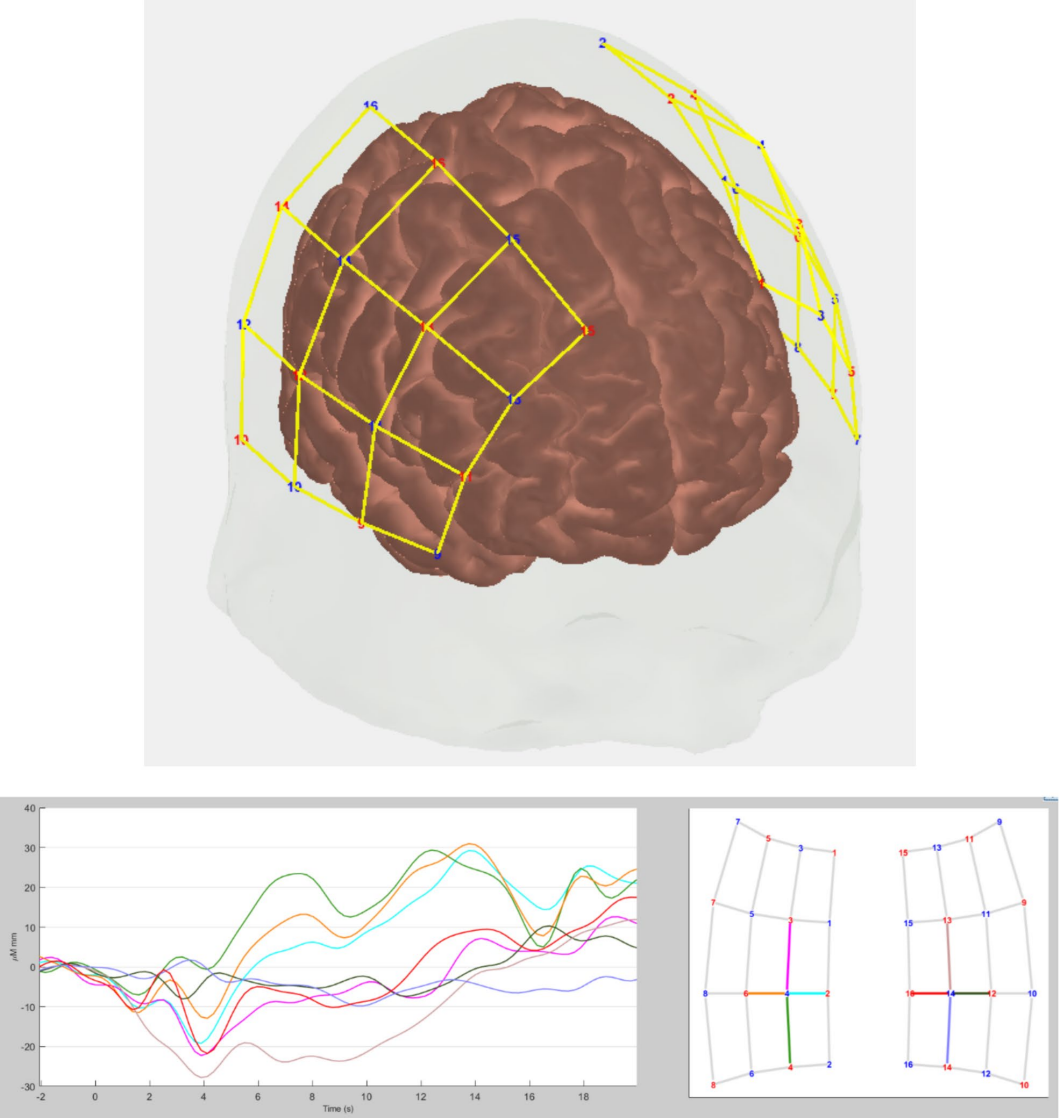
fNIRS Optode Montage and Haemodynamic Response Functions (HRFs). **Top Panel:** 3D rendering of the fNIRS optode montage over the cortical surface, showing source (red) and detector (blue) positions connected by yellow lines, representing measurement channels over bilateral frontal and motor cortices. The montage is designed to capture cortical haemodynamic activity in regions relevant to motor and cognitive functions. **Bottom Panel:** The left panel displays time series plots of empirical estimate of the HRFs (ΔHbO in mm) from block average (without assuming a model) from multiple channels during right-leg NMES showing larger response of oxygenated haemoglobin changes across time in left than right hemisphere. The right panel shows the 2D topographical layout of the channels, with specific channels highlighted, matching the plotted HRF curves. Colour-coded lines link the optodes to show the origin of each signal

The NIRSport2 fNIRS system (NIRx Medizintechnik GmbH) employed in this research offered flexible scalability featuring 16 light sources and 16 detectors to enable broad cortical coverage as shown in Fig. 2. Each module included high-intensity dual-tip LEDs capable of delivering up to 32 mW of illumination paired with advanced avalanche photodiode (APD) detectors capable of detecting signals as low as 5 femtowatts. To maintain signal quality, the system incorporated built-in automated optimization protocols, short-separation channels to help filter out extracerebral noise and integrated nine-axis accelerometers. Its lightweight and compact form factor approximately 900 grammes with dimensions of 162 × 125 × 60 mm^3^ allowed for secure placement and enhanced comfort for participants throughout the recording.

### fNIRS Data Analysis

Oxyhaemoglobin (HbO) and deoxyhaemoglobin (HbR) concentration changes were captured using the Aurora fNIRS Recording Software (NIRx Medical Technologies, LLC, Glen Head, NY, USA) and processed through the MATLAB-based NIRS Brain AnalyzIR Toolbox (MathWorks Inc., USA) specifically utilizing the *nirs.modules* pipeline. As described by [34], analysis is organized as a pipeline where each nirs.modules object processes data sequentially. For example, OpticalDensity converts raw light intensity into optical density, and BeerLambertLaw then estimates haemoglobin concentration changes (HbO/HbR). Then, pre-processing the fNIRS time series by first standardizing the sampling rate with nirs.modules.Resample then attenuating motion and systemic physiological noise using a Principal Component Analysis filter (*nirs.modules.PCAFilter)* that regresses out the highest-variance components whilst preserving stimulation-locked signals. The cleaned data are then passed to statistical models with autoregressive + iteratively reweighted least squares (AR-IRLS) being the recommended first-level GLM [34, 35]. AR-IRLS applies autoregressive prewhitening and robust iteratively reweighted least squares to reduce false positives under fNIRS-specific noise conditions. The resulting stats object contains brain activation estimates that can be further examined with higher-level modules (e.g. *nirs.modules.MixedEffects*) through the NIRS Brain AnalyzIR Toolbox.

#### Data Import

Data were loaded via *nirs.io.loadDirectory* organized by participant, posture condition (three seating positions), and levels of stimulation intensity.

#### Missing Data Management

Gaps in the dataset were addressed using the *FixNaNs* function (interpolates missing values using linear interpolation) to ensure continuity in the time series.

#### Event Synchronization

The onset of stimulation events was temporally aligned using the *ChangeStimulusInfo* module. A 1 Hz stimulation pattern was generated for the entire stimulation period initiated at the commencement of the geko™ device activation.

#### Demographic Integration

Participant information was incorporated into the dataset through the *AddDemographics* module.

#### Noise Filtering

Physiological artefacts were reduced by applying a band-pass filter with a frequency range of 0.01–2.0 Hz.

#### Motion Artefact Correction

Motion-induced noise was mitigated using Principal Component Analysis (*nirs.modules.PCAFilter, ncomp = 2*) in NIRS Brain AnalyzIR Toolbox [34].

#### Conversion to Optical Density

Raw light intensity data were converted into optical density values using the *nirs.modules.OpticalDensity* function.

#### Haemoglobin Concentration Computation and Heart Rate

HbO and HbR concentrations were derived using the modified Beer–Lambert Law via the *nirs.modules.BeerLambertLaw* module. We also computed total haemoglobin (HbT) as (HbO + HbR) and tissue oxygen saturation (StO_2_) as HbO/HbT. Then, the MSPTD (Multi-Scale Peak and Trough Detection) algorithm detected heartbeats in HbO signals by finding peaks and troughs at multiple time scales making it very robust against noise and signal variability [36]. The cardiac-related frequency cluster in fNIRS, typically around 1–1.5 Hz, appears in both oxyhaemoglobin and deoxyhaemoglobin signals but is more prominent in HbO due to its higher sensitivity to pulsatile blood volume changes [37]. First, the HbO signal is bandpass filtered (0.5–2.0 Hz with 2^nd^ order Butterworth) to isolate the individual heartbeat frequency cluster. Then MSPTD analysed the signal across scales to accurately detect each heartbeat peak. The time intervals between peaks were calculated to find the inter-beat intervals, and the heart rate (HR) is 60 divided by the beats per minute [38]. HR changes from baseline (no-stimulation condition) due to NMES were analysed separately for males and females where separate box plots were generated using MATLAB’s ‘*boxplot*’ function. The central line denotes the median whilst the lower and upper edges of the box represent the 25th and 75th percentiles respectively. Whiskers extend to the most extreme non-outlier data points, and outliers are displayed individually using a ‘ + ’ marker. Notches around the median provide a visual indication of the 95% confidence interval; non-overlapping notches suggest a statistically significant difference between medians.

### Outcome Measures

The main outcomes in this study were the beta (*β*) coefficients obtained through General Linear Model (GLM) analysis of the fNIRS data using the *nirs.modules.AR_IRLS* function. As described by Santosa et al. [34], the statistical modules in the NIRS Brain AnalyzIR Toolbox provide first- and higher-level statistical analysis of fNIRS data. At the first level, the most common approach is a GLM which relates measured haemoglobin signals to task design. The toolbox offers two main GLM solvers, the Ordinary Least Squares (OLS), and the AR-IRLS which is tailored for fNIRS noise. We used AR-IRLS that combines autoregressive (AR) prewhitening to handle serially correlated errors from physiology with iteratively reweighted least squares (robust regression) to down-weight motion-related outliers. This dual approach improved control of false positives compared to OLS. Beyond first-level models, the toolbox also supports mixed-effects (*nirs.modules.MixedEffects* function) models for group analysis that was used in this study. Together, these statistical tools allow both subject-level and group-level inference whilst accounting for fNIRS-specific noise characteristics.

To control for physiological confounders, the time series data for transcutaneous CO_2_ were resampled and included in the model as additional regressors using the *AddAuxRegressors* module. The resulting *β*-values represented the strength and direction of the association between experimental conditions and the observed haemodynamic responses (canonical HRF or double-gamma function used), namely, changes in HbO and HbR concentrations. The canonical HRF (double-gamma function) is the default basis set in the NIRS Brain AnalyzIR Toolbox. Canonical HRF models a haemodynamic response including an undershoot phase and is well suited for event-based designs. By estimating *β*-values for each measurement channel and condition, our analysis quantified the extent of neural activation elicited by NMES for event-based design. A positive *β*-value indicated an increase in haemoglobin concentration relative to baseline whilst a negative value reflected a decrease. This modelling approach enabled the identification of brain regions activated during NMES for event-based design whilst accounting for CO_2_-related physiological noise thereby enhancing interpretation of the underlying neural activity.

To interpret stimulation–response relationships in physiologically meaningful terms, pulse widths were grouped into two bins based on canonical chronaxie differences between peripheral fibre classes. Classic and modern electrophysiology sources report markedly shorter chronaxie for large myelinated A*α* motor fibres and longer chronaxie for smaller myelinated sensory fibres, whereas unmyelinated C fibres exhibit much longer chronaxie (~ 1–10 ms) [39–42]. Consistent with prior work [12], we binned pulse widths as (i) 35–140 µs (motor-fibre–weighted) and (ii) ≥ 200 µs (sensory-fibre–weighted). This binning is intended as a functional interpretation of excitability weighting rather than a definitive statement of selective recruitment since recruitment also depends on electrode placement, tissue impedance, and current amplitude.

### Statistical Analysis

#### Individual-Level Analysis

Haemodynamic responses (Boxcar HRF) for each participant were modelled using the *nirs.modules.AR_IRLS* function. Here, the boxcar HRF was used in block design to model sustained neural activation during continuous NMES unlike the canonical HRF which models event-related responses. A 30-second pre-stimulation baseline period was used for normalization following the methods described by Santosa et al. [34]. We assessed model fit quality at the single-subject level using the *nirs.modules.AR_IRLS* function. For each subject and channel, we extracted the regression coefficients, t-statistics, and p-values. To quantify fit quality we calculated, for each subject, the proportion of channels showing significant task effects after controlling the false discovery rate (Benjamini–Hochberg, *q* < 0.05). We also summarized the median absolute t-value and beta magnitude across channels. Residuals were visually inspected to confirm the absence of task-locked structure.

#### Group-Level Analysis

To evaluate effects across participants, a mixed-effects regression model was implemented using the *nirs.modules.MixedEffects* function. The *nirs.modules. MixedEffects* function fits models under normality assumptions using MATLAB’s fitlme function (Statistics and Machine Learning Toolbox, MathWorks Inc., USA) but does not test these assumptions directly [34]. Mixed-effects regression model accounted for fixed effects related to the experimental conditions (NMES-evoked response) and random effects due to inter-subject variability using robust fitting procedures. Outlier detection was handled via the *RemoveOutlierSubjects* module which flags individuals whose influence on the overall model exceeds a defined threshold (used cutoff = 0.05) based on statistical indicators like leverage and residual variance. Identified outliers were fully excluded from the group analysis (*allow_partial_removal = false*) to ensure consistency in comparisons across all conditions and contrasts. This method reduces the impact of atypical data points whilst maintaining the validity of the group-level results. We ran the group analysis with the *nirs.modules. MixedEffects* function (*formula: beta ~  − 1 + cond + (1|subject)*) and diagnostics enabled, storing a fitted mixed model per channel/condition to allow residuals QQ-plot based checks. These supplementary diagnostics were used to confirm that the group-level GLM fit was appropriate for subsequent inference.

Data Visualization: Topographical plots and statistical parametric maps (SPMs), were generated using the NIRS Brain AnalyzIR Toolbox [34]. Group-level SPMs were displayed on the standardized 10–20 schematic using the Clarke azimuthal projection (*probe.defaultdrawfcn = ’10–20 map’;*) as implemented in the NIRS Brain AnalyzIR toolbox [34] with significant channels (*q* < 0.05) shown. Also, violin plots were used to visualize the distribution of *β*-values across groups by combining a boxplot with a kernel density estimate (KDE). For each category, the KDE was computed using a Gaussian kernel to generate a smoothed probability density function, which was mirrored to form the violin shape. Medians, interquartile ranges, and 95% confidence limits were displayed within each violin. Plots were generated using Seaborn in Python providing a clear representation of both the central tendency and variability in the data.

### Computational Modelling

**Fig. 3:**
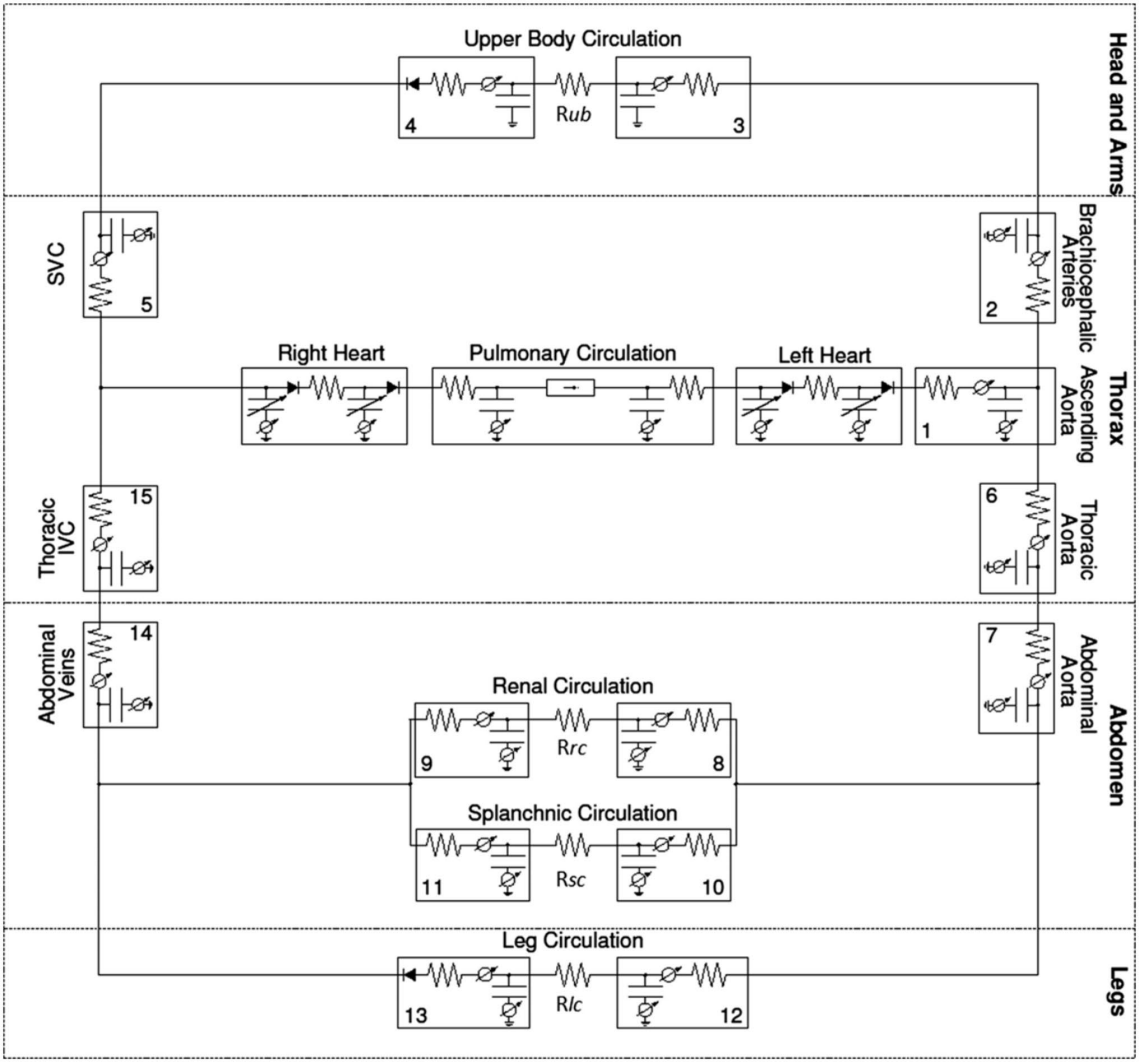
Circuit representation of the 21-compartment cardiovascular model divided into four regions-head/arms, thorax, abdomen, and legs. The labelled compartments are proximal aorta (1), brachiocephalic arteries (2), upper body precapillary (3) and postcapillary (4) compartments, superior vena cava (5), thoracic aorta (6), abdominal aorta (7), renal precapillary (8) and postcapillary (9) compartments, splanchnic precapillary (10) and postcapillary (11) compartments, leg precapillary (12) and postcapillary (13) compartments, abdominal veins (14), thoracic inferior vena cava (15), right atrium (16), right ventricle (17), pulmonary artery (18), pulmonary vein (19), left atrium (20), and left ventricle (21). Resistances represent vascular beds, while diodes represent heart and venous valves that ensure one-way blood flow, enabling simulation of systemic, pulmonary, and cardiac haemodynamics. Adapted from Diaz-Artiles et al. [43]

To understand the systemic impact from common peroneal NMES, we used a validated lumped-parameter cardiovascular model developed by Diaz-Artiles et al. [43] for simulating acute cardiovascular responses to gravitational (posture-related) and exercise (NMES intensity) conditions. The published model comprises a detailed 21-compartment representation of the cardiovascular system, encompassing systemic, pulmonary, and cardiac circulation. It integrates key physiological mechanisms, including gravitational effects, autonomic reflexes, and exercise-induced changes (see Fig. 3). The system is divided into four anatomical sections, i.e. the head and arms, thorax, abdomen, and legs with systemic compartments that include the proximal aorta, brachiocephalic arteries, upper and lower body precapillary and postcapillary compartments, venae cavae, renal and splanchnic circulations, and the heart. Venous valves are modelled using diodes in two venous compartments (upper body and leg) to ensure unidirectional flow, mimicking physiological behaviour.

During NMES, contracting muscles exert a mechanical pump effect by compressing venous vessels, which facilitates venous return. This muscle pump mechanism is implemented in the model by modulating external pressure applied to the venous leg compartment at 1 Hz matching the experimental NMES response [12]. The applied pressure waveform is described using a smooth, piecewise function, where the maximal pressure varies with NMES intensity. A separate centrifugal pump-related external pressure term in the model was set to zero in this study. The model also incorporates two key autonomic control mechanisms, i.e. the arterial baroreflex and the cardiopulmonary reflex. These are modelled as closed-loop controllers that respond to deviations from predefined pressure set-points by adjusting cardiovascular parameters via sympathetic and parasympathetic pathways. The arterial baroreflex is represented by a single lumped baroreceptor in the carotid sinus located 25 cm above the heart. It monitors the carotid sinus pressure computed as the aortic arch pressure minus the hydrostatic pressure gradient. The baroreflex adjusts heart rate, ventricular contractility, peripheral resistance, and venous tone across multiple regions. Similarly, the cardiopulmonary reflex uses the transmural right atrial pressure as its input variable. Both reflex systems use linear time-invariant filters to simulate sympathetic and parasympathetic dynamics and apply gain-specific control to modulate peripheral resistance and venous unstressed volumes in the upper body, kidneys, splanchnic circulation, and legs. Together, these reflexes ensure short-term regulation of blood pressure and venous return under conditions such as postural shifts and exercise [43]. During NMES (1 Hz for geko™ device), the model incorporated reduced leg vascular resistance to reflect metabolic vasodilation, muscle pump effects via time-varying external pressure to the leg veins, modified arterial pressure set point to account for exercise-induced sympathetic activation [43]. The model equations implemented in Simulink (Mathworks Inc. USA) were solved as a system of coupled first-order differential equations with physiological parameters derived from the published work [43]. This provided a computational framework for simulating 1 Hz NMES-induced haemodynamic changes across the cardiovascular system and understanding upstream effects on the cerebral perfusion.

## Results

**Fig. 4:**
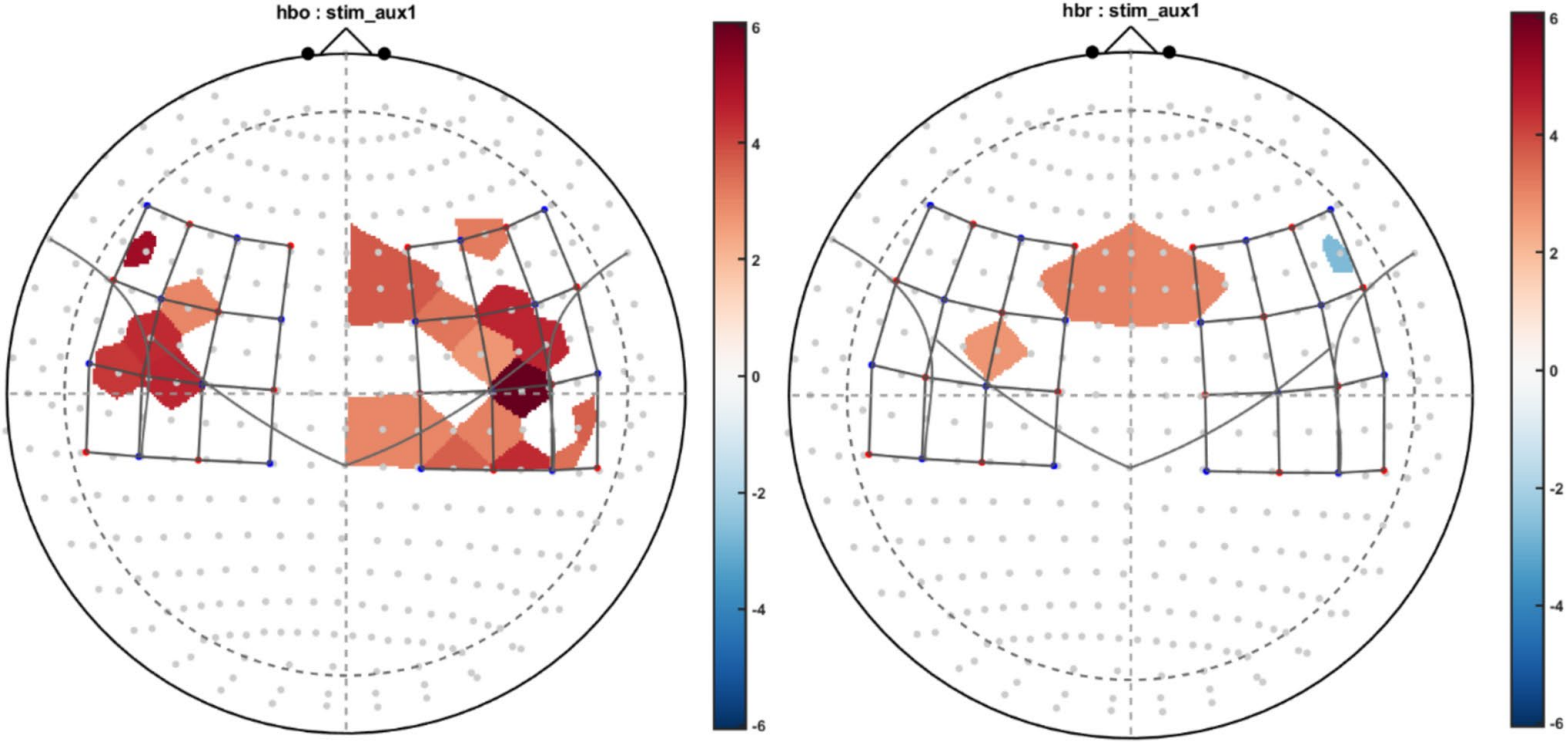
Topographical plots show the t-statistics of the GLM contrast (NMES-evoked > baseline) using canonical HRF for HbO, HbR on the probe layout. Only channels that passed the statistical threshold (*q* < 0.05 FDR) are rendered using Clarke azimuthal map projection

The clinical stroke study included 18 participants with a mean age of 76 years (standard error: 3 years; age range: 37 years). The sample comprised individuals with both left- and right- hemisphere strokes and consisted of 10 males (56%) and 8 females (44%). On average, measurements were taken 32 days after stroke onset (SE = 6 days; maximum interval = 125 days). Stroke severity at the time of data collection, assessed using the National Institutes of Health Stroke Scale (NIHSS), ranged from 0 to 10 with a mean score of 4 (SE = 1). Detailed baseline demographics, neuroimaging results, and vascular assessments are presented in Table 1. 84 data points including two subjects with the history of congestive cardiac failure (see Table 1) were excluded after being identified as outliers using the *RemoveOutlierSubjects* module from the NIRS Brain AnalyzIR Toolbox (see Methods). These data points exceeded a predefined threshold (*p* < 0.05) in the mixed-effects model to exert undue influence on model estimations. We excluded cases that showed systematic discrepancies likely arising from artefacts or physiological variability to minimize statistical bias and strengthen the validity of the group-level analyses. The final sample size of 16 participants reflected the outcome of this quality control process. At the single-subject level, an average of 0.17% (SD = 1.14%) of HbO/HbR channels (out of 96 per subject) were significant after FDR correction. The median absolute t-statistic across participants was 0.37, and the median absolute beta magnitude was 0.24 μM. These values indicate that significant activations were sparse at the single-subject level which is consistent with expectations for robust GLM analysis in fNIRS whilst accounting for NMES-evoked CO_2_-related physiological noise. Residual inspection did not reveal systematic structure, suggesting that the AR-IRLS model appropriately captured task-related variance whilst accounting for temporal autocorrelation. However, when results were aggregated at the group level, robust and spatially consistent activations were observed as shown in the topographical maps (Fig. 4).

**Table 1:** Baseline characteristics of 18 participants with a mean age of 76 years (standard error: 3 years; age range: 37 years)

Risk factor	Count (%)
History of hypertension	9 (50.0%)
History of diabetes	5 (27.8%)
History of stroke/transient ischaemic attack (TIA)	3 (16.7%)
Known atrial fibrillation (AF)	6 (33.3%)
History of congestive cardiac failure	2 (11.1%)
Current or ex-smoker	6 (33.3%)
Oxfordshire community stroke project (OCSP) classification	
Total anterior circulation stroke (TACS)	7 (38.9%)
Partial anterior circulation stroke (PACS)	4 (22.2%)
Lacunar stroke (LACS)	3 (16.7%)
Posterior circulation stroke (POCS)	4 (22.2%)
Location of the main lesion	
Cortical	9 (50.0%)
Others	9 (50.0%)
Size of the infarct	
> 5 cm	5 (28.0%)
1.5–5 cm	9 (50.0%)
< 1.5 cm	4 (22.0%)
Ipsilateral internal carotid artery (ICA) Stenosis	
< = 50%	16 (89.0%)
> 50%	2 (11.0%)
Number of intracranial artery stenoses (ICAS)	
None	4 (22.2%)
1	11 (61.1%)
> 1	3 (16.7%)

Figure 4 shows topographic SPMs of NMES-evoked changes in HbO and HbR concentrations (using canonical HRF) at the group level in the final sample of 16 participants (using boxcar HRF in Fig. S1, Online Resource SupplementaryFigures.zip). Significance was assessed using a false discovery rate threshold (*q* < 0.05). Warm colours indicate positive task-related changes and cool colours negative changes which uses a Clarke azimuthal map projection [34]. NMES produced widespread HbO (and HbT in Fig. S6, Online Resource SupplementaryFigures.zip) increases with smaller, more focal HbR effects (and negligible StO_2_ changes in Fig. S6, Online Resource SupplementaryFigures.zip), consistent with increased global perfusion during stimulation [12]. After adjusting for the NMES-evoked CO_2_ response (physiological confounder) the SPMs likely revealed two components, i.e. a global neurovascular response primarily evident in HbO and a metabolism-linked effect evident in HbR. The pronounced neurovascular (HbO, HbT) component likely also reflects post-stroke autonomic dysregulation [30]. Group maps showed bilateral HbT increases over the sensorimotor coverage consistent with an NMES-induced rise in total haemoglobin/blood volume (Fig. S6, Online Resource SupplementaryFigures.zip). In contrast, StO_2_ yielded no surviving channels, indicating negligible change in tissue oxygen saturation. Together, these findings suggest that the dominant NMES effect is volumetric (perfusion/blood volume) rather than a robust shift in oxygen saturation (StO_2_).

NMES-evoked autonomic effects likely introduced systemic artefacts into fNIRS complicating the isolation of purely neuronal effects [44]. Therefore, we incorporated concurrent measures of systemic physiology [45], specifically transcutaneous CO_2_ response and MSPTD to detect heartbeats, to help disentangle systemic effects [46] through computational modelling (Fig. 3) [43]. To verify mixed-effects regression model assumptions, we inspected group residuals (Fig. S7, Online Resource SupplementaryFigures.zip). Model diagnostics indicated approximately normal residuals with no major deviations on the QQ plot and a symmetric, zero-centred histogram supporting the Gaussian error assumption. Together, these diagnostics support the adequacy of the mixed-effects GLM for inference.

**Fig. 5:**
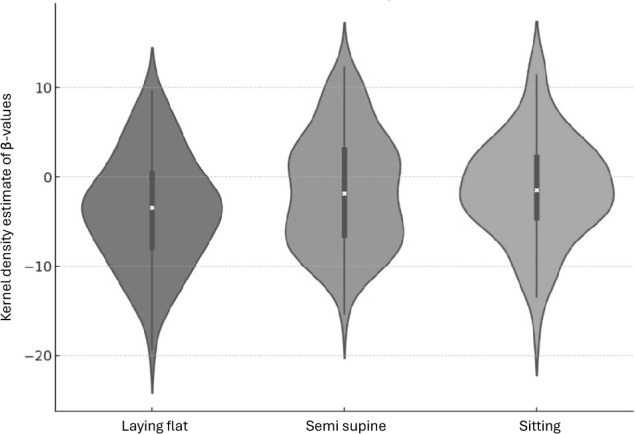
Effect of three body positions (laying flat or supine, semi-supine, and sitting or seated upright) on the beta coefficients (*β*-values) from GLM analysis of oxyhaemoglobin response using boxcar HRF

Figure 5 presents violin plots illustrating the influence of body posture (supine, semi-supine, and seated upright) on HbO beta values derived from the GLM analysis. Fig. S1 (Online Resource SupplementaryFigures.zip) shows the corresponding linear mixed-effects model from the NIRS Brain AnalyzIR Toolbox, adjusted for covariates, revealing a slight upward trend in activation with upright postures. Importantly, this upward trend must be interpreted in light of the distinction between functional and systemic components of the fNIRS signal where systemic physiology (e.g. global haemodynamic fluctuations and posture-related vascular changes) can contaminate fNIRS measurements likely linked to the autonomic dysfunction post-stroke [30].

**Fig. 6:**
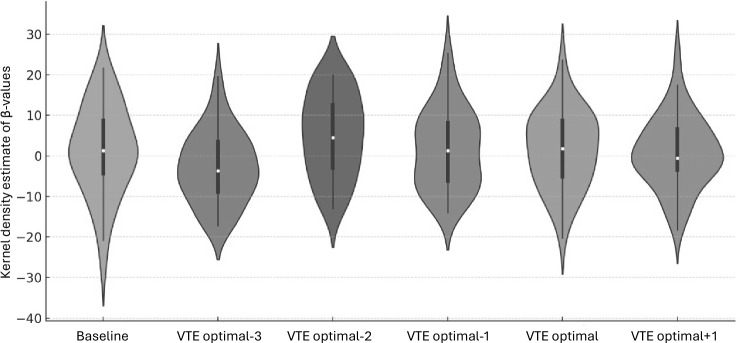
Effect of the NMES levels one level above optimal (VTE optimal + 1), the optimal level (VTE optimal), and three levels below optimal (VTE optimal–1, VTE optimal–2, VTE optimal–3) on the beta coefficients (*β*-values) from GLM analysis of oxyhaemoglobin response using boxcar HRF

Figure 6 shows violin plots illustrating how different levels of NMES intensity (one level above optimal, optimal, and three levels below optimal) influence HbO beta values. Fig. S2 (Online Resource SupplementaryFigures.zip) presents the corresponding linear mixed-effects model from the NIRS Brain AnalyzIR Toolbox revealing a slightly positive relationship with stimulation intensity. These results should again be interpreted with caution as fNIRS signals comprise both functional and systemic components and systemic physiology may contaminate the measurements likely linked to the autonomic dysfunction post-stroke [30].

**Fig. 7:**
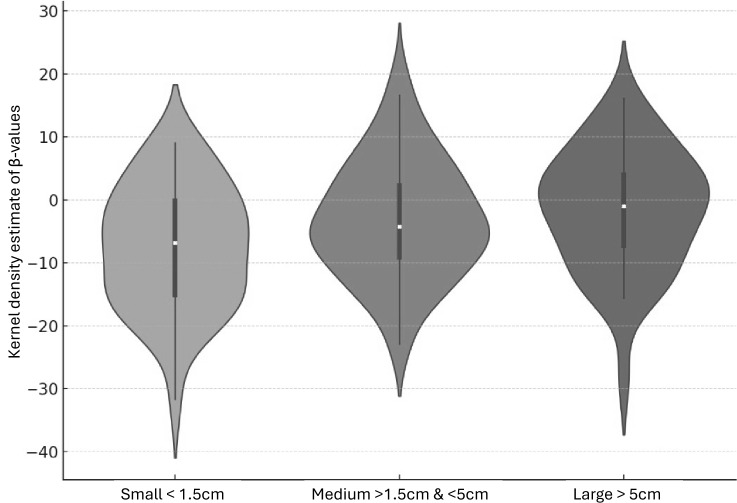
Effect of the size of the largest lesion (Small lesion size: < 1.5 cm, medium lesion size: 1.5–5 cm, large lesion size: > 5 cm) on the beta coefficients (*β*-values) from GLM analysis of oxyhaemoglobin response using boxcar HRF

Figure 7 shows violin plots depicting the relationship between lesion size categorized as small (< 1.5 cm), medium (1.5–5 cm), and large (> 5 cm) and HbO beta values. Fig. S3 (Online Resource SupplementaryFigures.zip) presents the mixed-effects analysis, showing that participants with larger lesions exhibited higher HbO beta values, suggesting stronger haemodynamic responses. This trend may reflect disrupted autoregulation or elevated metabolic demand in larger affected brain regions but it must also be interpreted in the context of systemic contributions to fNIRS signals likely associated with greater autonomic dysfunction after stroke in patients with larger lesions [30]. Computational modelling was therefore applied (Fig. 3) [43] to better understand systemic influences and to provide a physiological interpretation.

**Fig. 8:**
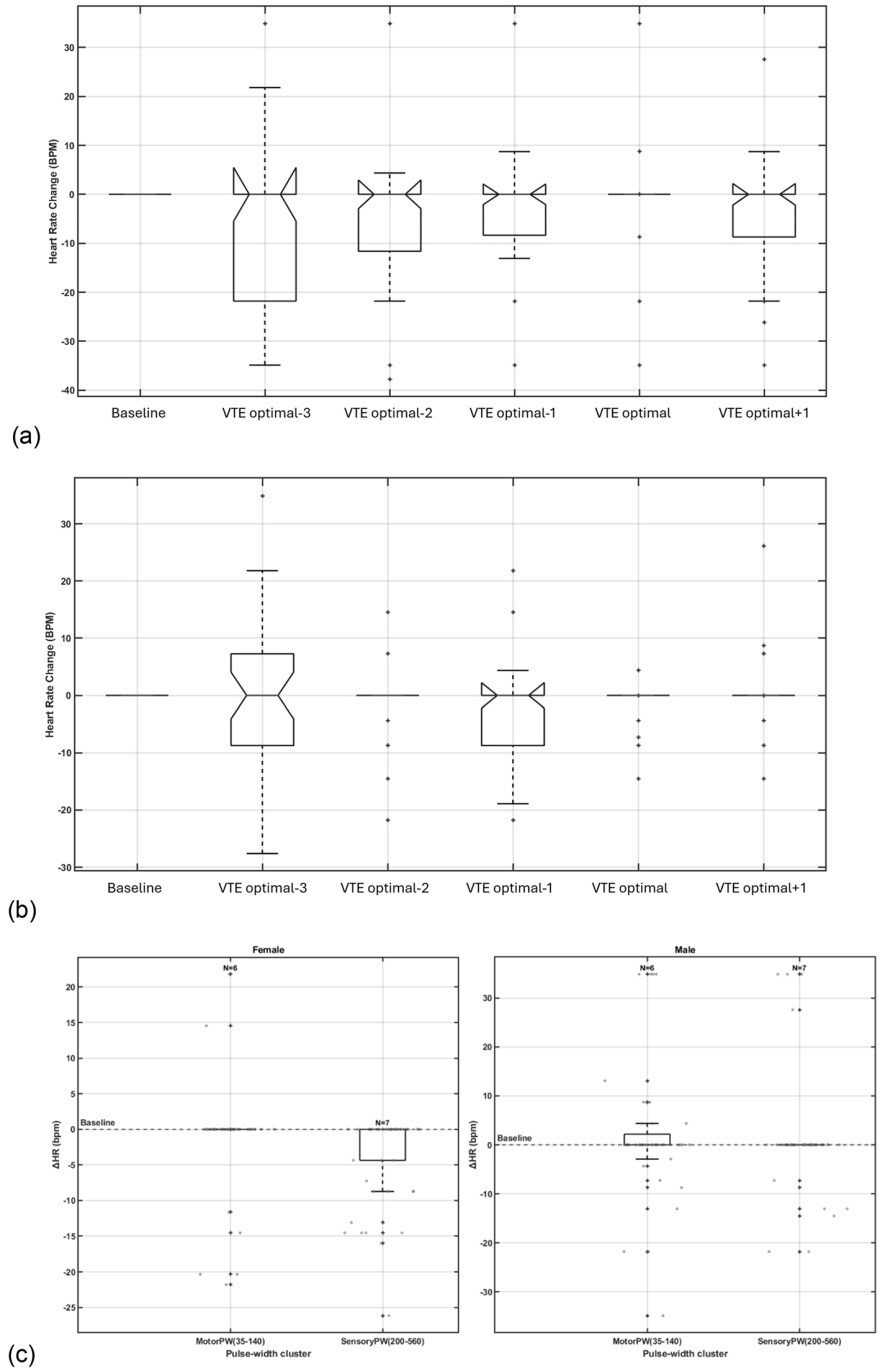

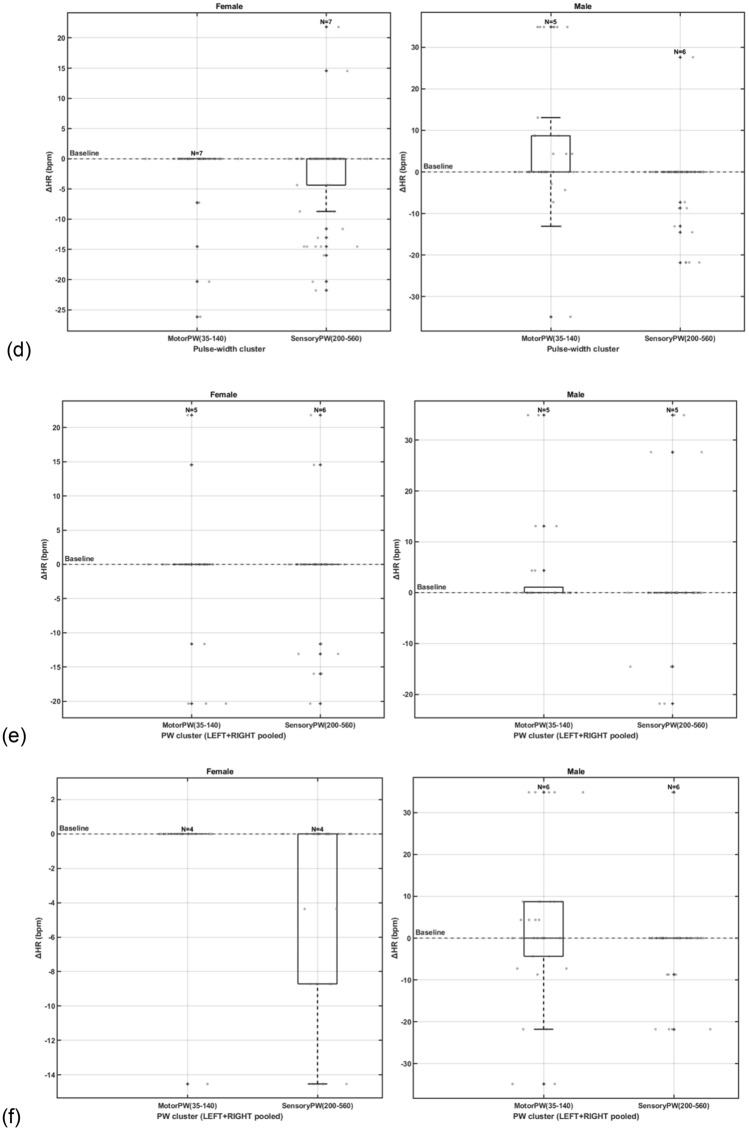
Heart rate changes from baseline (no-stimulation condition = 0) due to NMES application shown separately for males (**a**) and females (**b**) using box plots. Boxes show the median (central line), interquartile range (25th–75th percentiles), and whiskers extend to non-outlier data. Outliers are marked with ‘ + ’. Notches indicate the 95% confidence interval for the median. **c** Sex-stratified change-from-baseline of heart rate (ΔHR) plotted against left-leg CPN stimulation pulse-width fibre-class group. Pulse widths were binned to reflect canonical chronaxie ranges where short pulse widths (35–140 µs) were grouped as motor-fibre–weighted (shorter chronaxie typical of large myelinated A*α* motor axons) whereas longer pulse widths (≥200 µs; including 200, 280 µs @27 mA, 280/400 µs @38 mA, 400, 560 µs @54 mA) were grouped as sensory-fibre–weighted (longer chronaxie favouring smaller myelinated A*β*/Aδ afferents). Points represent individual trials; boxplots summarize the distribution across trials (median, interquartile range, whiskers). Group labels show the number of distinct participants contributing data. Δ values > 0 indicate an increase relative to the no-stimulation baseline within the same posture. **d** Same analysis as Fig. 8c but using right-leg CPN stimulation pulse-width fibre-class grouping. Heart rate changes (ΔHR) relative to the no-stimulation baseline (StimulationLevel = 1) during 1 Hz CPN NMES are shown for sitting-upright (**e**) and laying flat (**f**) postures. Stimulation conditions were grouped by pulse width into motor-fibre–weighted (35–140 µs) and sensory-fibre–weighted (≥ 200 µs) categories. Data are shown separately for females (left panels) and males (right panels). Boxplots represent the median and interquartile range, whiskers indicate non-outlier extrema, and individual points denote trial-level observations; annotations indicate the number of distinct participants contributing to each condition

Figure 8 illustrates heart rate (HR) changes from baseline (no-stimulation condition = 0) to NMES condition, presented separately for males (Fig. 8a) and females (Fig. 8b) using boxplots. The central line represents the median, box edges the 25th and 75th percentiles, whiskers the most extreme non-outlier data points, and outliers are marked with a ‘ + ’. Notches around the median indicate the 95% confidence interval, where non-overlapping notches denote statistically significant differences between medians. To examine the influence of neural recruitment profile on cardiovascular responses, stimulation conditions were stratified by pulse width into two mechanistically motivated categories, i.e. a motor-fibre–weighted range (35–140 µs), corresponding to shorter chronaxie values typical of large myelinated A*α* motor axons, and a sensory-fibre–weighted range (≥ 200 µs), which preferentially engages smaller myelinated sensory afferents. Baseline-referenced changes in heart rate (ΔHR) were then compared between these categories. Across the cohort, the sensory-fibre–weighted stimulation range demonstrated a systematically different distribution of ΔHR relative to the motor-fibre-weighted range, indicating that pulse-width-dependent fibre recruitment influences the direction and magnitude of cardiac autonomic effects (Fig. 8c and d). Sex-stratified analyses revealed that this pattern was not uniform with female participants predominantly exhibited reductions in ΔHR during sensory-fibre–weighted stimulation whereas males showed a more variable or attenuated response. Moreover, across postures (Fig. 8e and f), sensory-fibre–weighted stimulation was associated with a systematic shift towards heart rate reduction in females particularly in the sitting-upright posture. In the laying flat posture, ΔHR distributions were more negative overall consistent with reduced orthostatic load but sex-specific differences persisted. These findings indicate that pulse-width-dependent afferent recruitment interacts with biological sex and posture to shape autonomic cardiovascular responses consistent with known sex differences in cardiovagal–sympathetic balance and orthostatic regulation.

Figure S4 (Online Resource SupplementaryFigures.zip) shows how 1 Hz NMES modulates the cardiac component (0.5–2.0 Hz) of the HbO signal, derived from fNIRS, using a stabilization diagram [37]. This system identification method isolates dominant dynamic modes, i.e. frequency components and their damping characteristics within haemodynamic time series. Fig. S5 (Online Resource SupplementaryFigures.zip) demonstrates that transcutaneous partial pressure of carbon dioxide levels remained relatively stable during NMES with only small gradual changes observed in some subjects indicating minor interindividual variability. Here, it should be acknowledged that our current understanding of how NMES simultaneously evokes both functional haemodynamic responses and systemic alterations in global cerebral blood circulation remains incomplete. Even with a comprehensive theoretical model of blood circulation, CrCP, key parameters such as body tilt angle/orthostatic stress, autonomic nervous system activity, and CO_2_ concentration would still require measurement using methods beyond fNIRS [46]. Nonetheless, changes in different aspects of blood circulation can be linked to distinct haemodynamic outcomes. Here, blood flow velocity and volume are critical determinants and when these parameters vary across different vascular compartments (arteries, arterioles, capillaries, venules, and veins) they give rise to distinguishable haemodynamic modalities that can be separated using modal analysis [47]. Very slow oscillations in cerebral haemodynamics localized to the capillaries of metabolically active cortical regions are characterized by a negative correlation between HbO and HbR changes whilst low-frequency oscillations have a mix systemic fluctuations arising in extraparenchymal cerebral vasculature that are typically associated with a positive correlation between HbO and HbR changes and may be separated [46, 47].

**Fig. 9:**
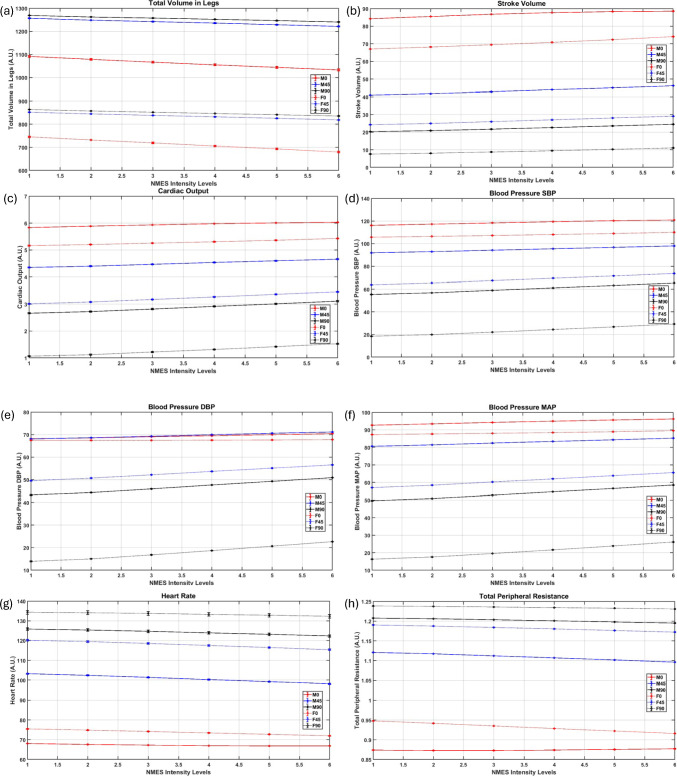
Effects of calf muscle stimulation and postural tilt on haemodynamic parameters across gender. Plots show comparisons for males at 0° (M0), 45° (M45), and 90° (M90) tilt, and females at 0° (F0), 45° (F45), and 90° (F90) tilt. **a** Total leg volume decreases progressively with increasing tilt angle, with males exhibiting slightly higher volumes than females. **b** Stroke volume reduces significantly with tilt, particularly in females at 90°, indicating greater preload dependency. **c** Cardiac output declines with upright tilt but is better preserved in males. **d** Systolic blood pressure remains relatively stable, with a modest increase at 90° tilt across both sexes. **e** Diastolic blood pressure shows a marked increase with tilt, greater in males. **f** Mean arterial pressure increases from supine to upright postures, maintaining cerebral perfusion. **g** Heart rate rises significantly at higher tilt angles, with females displaying a steeper increase than males. **h** Total peripheral resistance rises sharply with posture tilt, with females showing a greater relative increase compared to males

Figure 8 suggests that NMES evokes systemic alterations in global cerebral blood circulation by engaging intrinsic cardiovascular regulatory mechanisms differently in males and females [48] underscoring the need for personalized treatment approaches. To further examine these sex-specific systemic effects, we investigated the impact of rhythmic lower-limb muscle pump activation combined with graded postural tilt (0, 45, and 90°, corresponding to supine, semi-supine, and seated positions) on cardiovascular parameters using a lumped-parameter cardiovascular model [43]. Computational findings (Fig. 9) revealed progressive reductions in total leg volume, stroke volume, and cardiac output with increasing tilt, reflecting gravitational challenges to venous return. Males maintained higher absolute stroke volume and cardiac output across all tilt conditions likely due to greater preload capacity whereas females exhibited a steeper decline in these parameters accompanied by compensatory increases in heart rate and total peripheral resistance. These patterns are consistent with known gender-specific autonomic differences in which females rely more heavily on tachycardia and vasoconstriction to preserve mean arterial pressure during orthostatic stress [32]. Systolic and diastolic pressures rose moderately with tilt whilst mean arterial pressure was preserved across sexes reflecting intact baroreflex-mediated compensation. Collectively, these results indicate that NMES-induced muscle activation alters circulatory dynamics in a gender-dependent manner with distinct regulatory strategies that require personalized treatment approaches [24].

## Discussion

The RETRAIN Phase 1 study provided preliminary yet compelling evidence that 1 Hz NMES applied to the common peroneal nerve can positively modulate cortical oxygenation in stroke survivors. Our approach combining experimental data, computational modelling, and clinical observations demonstrates that NMES enhances cortical oxygenation with modulation by body posture and lesion characteristics. Consistent with prior findings [12, 25], 1 Hz NMES appears to augment cerebral haemodynamics likely through enhanced venous return, increased cardiac preload, and hypercapnia-induced cerebral vasodilation with notable interindividual variability influenced by gender. Our computational modelling confirmed the gender-specific cardiovascular adaptations (Fig. 9) that were partly observed experimentally (Fig. 8) [28]. In males, low-intensity NMES (e.g. VTE optimal–3) induced pronounced bradycardia likely reflecting autonomic modulation such as enhanced parasympathetic tone. This effect diminished near optimal stimulation levels and remained modest above them. In females, low-intensity NMES elicited smaller median reductions in heart rate with greater variability; the larger interquartile range and more frequent outliers in females indicate greater heterogeneity in physiological responses to NMES. Then, pulse-width binning was designed to connect device settings to established excitability differences across peripheral fibre classes. Because chronaxie is shorter for large myelinated A*α* fibres and longer for A*β*/Aδ fibres, increasing pulse-width shifts the stimulus towards regimes that can more readily excite smaller-diameter myelinated afferents at a given perceived intensity, whilst very long pulse widths (ms range) would be required for strong weighting towards unmyelinated C fibres. Therefore, any systematic shift in ΔHR outcomes between the short- and long- pulse-width groups is consistent with a change in relative recruitment weighting amongst fibre classes with altered reflex/autonomic contributions whilst acknowledging that selective recruitment cannot be inferred from pulse-width alone. In this study, current amplitude and electrode geometry were constrained by the geko^TM^ Electro-Stimulation Device and its protocol requirements, and thus their pulse widths (35, 50, 70, 100, 140, 200, 280 μs @27 mA, 280 & 400 μs @38 mA, 400, 560 μs @54 mA) served as a pragmatic, physiologically anchored proxy to probe the recruitment regime. The larger HR reduction observed in females during sensory-weighted pulse widths may reflect sex differences in autonomic regulation and reflex cardiovascular control, which are known to be modulated by sex hormones and to influence cardiovagal and sympathetic balance. Whilst the present experiment does not isolate neural pathways, convergent evidence from autonomic physiology and afferent neuromodulation indicates that peripheral afferent stimulation can bias central autonomic outflow in a direction consistent with reduced heart rate [49, 50] in the absence of compensatory increases in heart rate and total peripheral resistance modelled in Fig. 9. Future work should normalize electro-stimulation dose to sensory/motor thresholds and formally test these interactions in mixed-effects models controlling for posture and baseline HR. Our findings in Fig. 8c–f underscore gender-specific differences in autonomic cardiovascular regulation during electrical peripheral nerve stimulation and suggest that optimizing NMES in combination with body posture may require tailoring to gender-based cardiovascular physiology to maximize therapeutic benefit. Here, our findings support development of a clinically interpretable machine learning framework for stratifying NMES responsiveness with larger validation studies needed [24].

Figure 4 shows group-level GLM maps for the NMES condition (stim_channel1), plotted separately for HbO, HbR (HbT and StO_2_ in Fig. S6 Online Resource SupplementaryFigures.zip) on the 2-D probe layout. Across participants, HbO exhibited the most robust and spatially extended responses with multiple clusters over the sensorimotor coverage of the probe. HbT (total haemoglobin) showed a similar spatial pattern to HbO consistent with increases in regional blood volume/flow during NMES. HbR effects were weaker and more focal than HbO/HbT which is common in fNIRS because HbR has smaller amplitude and lower SNR. The direction of HbR change was small relative to HbO and varied across channels. StO_2_ maps showed no consistent effect which is also typical because StO_2_ is a ratio measure and tends to be less sensitive to brief, localized NMES responses. Overall, these SPMs indicated that NMES evoked increases in cortical oxygenation and blood volume in the regions sampled by the probe with the expected dominance of HbO/HbT over HbR and minimal StO_2_ changes. This pattern is consistent with neurovascular coupling in sensorimotor networks engaged by somatosensory afference during NMES [12].

1 Hz NMES-induced cerebral oxygenation responses were potentiated in the seated upright posture (Fig. 5, Fig. S1) where gravitational pooling of venous blood poses a challenge to preload maintenance. 1 Hz NMES likely counteracts these gravitational pooling effects, stabilizing central haemodynamics and enhancing cerebral perfusion consistent with prior reports that posture strongly influences cerebrovascular dynamics [33, 51]. Larger infarct volumes (> 5 cm) were associated with more pronounced cortical haemodynamic responses (Fig. 7, Fig. S3) suggesting impaired autoregulation or increased neurovascular response in line with evidence of altered neurovascular coupling in severe stroke [52, 53]. fNIRS provided real-time, non-invasive monitoring of superficial cortical haemodynamics, and despite its limited depth sensitivity, remains valuable for bedside assessment when combined with rigorous noise reduction and physiological confounder controls. Importantly, this RETRAIN Phase 1 study is amongst the first to integrate cardiovascular computational modelling with NMES-evoked cerebral perfusion changes, demonstrating through a lumped-parameter model that NMES can safely augment preload and cardiac output via intact baroreflex and cardiac function excluding two participants with heart failure.

Several limitations should be acknowledged, including the lack of evidence on the long-term effects of NMES treatment, for example in SHS and post-stroke dementia [54]. First, the modest sample size limits the generalizability of these findings, and although statistical rigour was improved by outlier removal, physiologically relevant variability may have been excluded. Second, the lack of a sham control arm constrains interpretation as NMES-specific effects cannot be fully separated from placebo or expectancy-driven influences. Third, fNIRS measurements are confined to the superficial cortex and extracerebral tissues (scalp and meninges), and cerebral perfusion is inferred indirectly from changes in oxy- and deoxy-haemoglobin rather than measured directly. In contrast, optical methods such as diffuse correlation spectroscopy (DCS) and speckle contrast optical spectroscopy (SCOS) provide direct flow-based measures of cortical haemodynamics. Notably, DCS/SCOS addresses a key limitation of fNIRS by offering greater specificity to blood flow and reducing contamination from extracerebral and systemic signals. Fourth, inter-individual variability in autonomic regulation particularly impaired baroreflex sensitivity post-stroke which was not measured may have influenced NMES responses. Finally, whilst NMES acutely enhanced cortical oxygenation, its long-term effects on neuroplasticity and functional recovery remain to be established.

Future work should address some of the limitations and explore combining fNIRS with advanced flow-based modalities such as DCS or SCOS [55] to enable non-invasive monitoring of critical closing pressure (CrCP) and arteriole compliance [56]. A recent study compared three non-invasive optical blood flow measurement techniques that are DCS, interferometric diffusing wave spectroscopy (iDWS), and SCOS using concurrent in vitro and in vivo experiments [57]. DCS estimates microvascular blood flow by analysing temporal fluctuations in near-infrared light scattered by moving red blood cells. iDWS builds on this principle by incorporating interferometric detection which improves sensitivity and signal-to-noise ratio. SCOS, in contrast, quantifies flow by analysing speckle contrast patterns generated by coherent light, providing a flow-sensitive measure with high temporal resolution. In flow phantom experiments, iDWS and DCS produced equivalent absolute flow values whilst iDWS and SCOS achieved substantially higher signal-to-noise ratios than DCS. SCOS also showed relative flow changes consistent with both DCS and iDWS. In vivo cuff occlusion experiments confirmed agreement across all three techniques for relative flow dynamics though DCS reported a higher flow pulsatility index than iDWS or SCOS. Collectively, these findings demonstrate that SCOS offers improved precision for pulsatile waveform assessment highlighting the potential for future optical blood flow monitoring. SCOS, when integrated with arterial blood pressure (ABP) waveforms can replace transcranial Doppler–derived CrCP [58] whilst also providing unique estimates of arteriole compliance. To characterize the haemodynamic properties of the microvascular unit, the arteriole compartment and the pre-capillary sphincter can be modelled using its lumped-parameter electrical analogue (Fig. S4 in Online Resource SupplementaryFigures.zip) to enhance the current model. In the physiological representation, blood flow enters the arteriole compartment under an input arterial pressure and passes through to the pre-capillary sphincter where the effective lumen area and the CrCP determine outflow towards the capillary compartment. To facilitate quantitative analysis, one can express this system as an equivalent electrical circuit in which the arteriolar compartment is represented by a compliance in parallel with a resistance whilst the pre-capillary sphincter is modelled as a variable resistance in series. This formulation allowed us to capture both the pressure–flow relationship across the arteriole and the modulatory role of the pre-capillary sphincter in determining capillary perfusion pressure. Data-driven modal analysis [59, 60] can identify the components that span cardiac (~ 0.6–2 Hz), respiratory (~ 0.15–0.4 Hz), Mayer/sympathetic (~ 0.07–0.15 Hz), myogenic (~ 0.05–0.15 Hz), neurogenic (~ 0.02–0.05 Hz), and endothelial/metabolic (< ~ 0.02 Hz) bands; however, in output-only settings with additional unmeasured periodic inputs, subspace-based system identification remains consistent but modal estimation may be biased by periodic excitation. To address this, periodic components can be estimated using a non-stationary Kalman filter and removed via orthogonal projection prior to parameter estimation, thereby improving recovery of the underlying structural (physiological) modes and enhancing interpretability of stabilization analyses. Then, diameter-dependent viscosity (Fåhræus–Lindqvist/phase separation effects) can modulate shear stress and microvascular impedance, enabling endothelial shear-feedback to shape low-frequency vasomotion [47, 61, 62]; however, oscillations can also arise purely from nonlinear rheology in microvascular networks [63]. Thus, haematocrit-related disorders can modify resistance and shear and, secondarily, influence vascular tone, producing indirect shifts in the effective CrCP rather than determining it directly. For instance, the reduction in 0.01–0.02 Hz power was more pronounced in older participants with type-2 diabetes mellitus and cognitive impairment, consistent with attenuated endothelial/NO-mediated vasomotion [64]. Such dampening could be compatible with increased microvascular resistance and a higher ‘effective CrCP’, conditions that favour intermittent or reduced capillary perfusion at low driving pressures. Future research should include randomized, sham-controlled trials in larger and more diverse stroke cohorts. Multimodal validation for example, integrating fNIRS with SCOS could improve bedside monitoring of cerebral perfusion, vascular tone, and autoregulatory capacity, providing a physiologically relevant alternative to invasive intracranial pressure monitoring [65]. Here, individualisation of NMES parameters represents a testable hypothesis for subsequent studies [24]. Specifically, stimulation frequency and phase optimisation could be guided by real-time physiological monitoring including heart rate (HR), blood pressure (BP), end-tidal CO_2_, and cardiac output integrated with concurrent cerebral metrics (e.g. cortical oxygenation, perfusion). Such an approach would enable closed-loop adjustment of stimulation parameters to maximise haemodynamic support. We propose a hierarchical control architecture for safe dosing comprising, (i) a rapid safety layer enforcing haemodynamic constraints using beat-to-beat (or short-window) HR and intermittent/continuous BP to prevent hypotension or tachycardia; (ii) a confound-control layer incorporating transcutaneous or end-tidal CO_2_ to contextualise cerebral oxygenation changes and mitigate CO_2_-driven artefacts; and (iii) a slower efficacy layer utilising fNIRS-derived HbO/HbT and/or SCOS-derived perfusion metric (with short-separation regression and systemic-noise correction) for optimisation. Finally, studies assessing functional outcomes such as motor recovery, cognition, and quality of life are needed to establish the clinical utility of NMES in stroke rehabilitation.

In conclusion, NMES represents a promising adjunct therapy capable of enhancing peripheral and cerebral circulation during the vulnerable phase of stroke recovery. RETRAIN Phase 1 study provided mechanistic in-silico modelling and a synthetic patient cohort to study 1 Hz NMES therapy after ischaemic stroke. Using multimodal monitoring (NIRS, SCOS, ABP), our study in the future can track cerebrovascular dynamics including CrCP and arteriole compliance that are indices of vascular tone and autoregulation [6]. These signals can calibrate a “digital twin” cardiovascular-cerebrovascular model and a closed-loop model predictive control system then optimizes NMES in near real time to sustain cerebral perfusion whilst maintaining systemic safety. Furthermore, by incorporating stroke-specific factors [24], our stratification framework enables a more biologically informed and individualized therapeutic approach. Our multivariable model (AUROC = 0.723) retained 13 independent predictors including cortical lesion location (OR = 1.66) and atrial fibrillation (OR = 1.28) [24]. Additional mechanistic contributors likely include baseline arterial compliance, endothelial function, haematocrit and blood viscosity, venous capacitance, medication exposure (e.g. *β*-blockers, antihypertensives), lesion burden and topography, and coexisting cardiac comorbidities (e.g. atrial fibrillation, chronic cardiac failure). Consideration of these interacting cerebrovascular and systemic determinants provides a physiologically grounded basis for precision titration of NMES. Moreover, integration of these clinically relevant variables enhances responder stratification and underpins a pathophysiologically informed, individualized therapeutic framework that mechanistically links cardiovascular support with cerebral perfusion to bridge neuroprotection and early neurorecovery in the acute post-stroke period. Importantly, the brain–heart interplay in the critical post-stroke period creates a dual risk as cerebral ischaemia is worsened by reduced cardiac output and autonomic dysregulation. Delayed SHS onset (10–30 days) nearly doubled 90-day mortality compared with early onset [3]. Overall, SHS markedly increases post-stroke mortality particularly when onset is delayed which may be prevented with neurostimulation.

## Data Availability

The neuroimaging results analyzed in the current study and the MATLAB code can be accessed at https://github.com/Siagnos/CV-Model-NMES.
